# Supplementary material for: The C-Terminal RRM/ACT Domain Is Crucial for Fine-Tuning the Activation of ‘Long’ RelA-SpoT Homolog Enzymes by Ribosomal Complexes
Source: Front Microbiol. 2020 Feb 28;11:277. doi: 10.3389/fmicb.2020.00277 (PMC7058999; doi:10.3389/fmicb.2020.00277)
Supplement: Supplementary file 1 [file Data_Sheet_1.PDF]

Supplementary Material

1.1 Construction of *E. coli* plasmids

Mutations in RelA$^{\Delta RRM}$ ORF were introduced using two step PCR (Ho et al., 1989). Specifically, primers MR3 and MR4 were used to introduce H432E, MR5 and MR6 to introduce R629E, MR7 and MR8 to introduce C612A/C613A; primers MR1 (forward) and MR2 (reverse) were universally used to amplify the PCR products. The resultant linear fragments were digested with *Bam*HI and *Xho*I enzymes (ThermoScientific) and cloned into pNDM220 (Gotfredsen and Gerdes, 1998) plasmid cut with the same restriction enzymes.

1.2 Construction of *B. subtilis* strains and plasmids

To construct the strain NBS1437 [*trpC2 relQ::tet relP::spcR*], first, two ≈500 nucleotide-long DNA fragments were amplified by PCR using wt168 genomic DNA as a template: one located upstream of the *relQ* ORF (primers: NOP7 and NOP8), another located downstream of the *relQ* ORF (primer, NOP11 and NOP12). Second, the tetracycline resistance marker (*tet*) of pDG1515 (Guerout-Fleury et al., 1995) was amplified by PCR using the primers NOP9 and NOP10. All three PCR products described above were used simultaneously as the template for PCR amplification using primers NOP7 and NOP12. The resulting long fragment was used to transform strain 168 (*trpC2*). *relQ* deletion mutants were selected by tetracycline resistance, yielding the NBS1393 [*trpC2 relQ::tet*] strain. Chromosomal DNA extracted from strain RIK908 [*trpC2 relP::spcR*] was used to transform the strain NBS1393 and selected by spectinomycin resistance yielding the strain NBS1437.

To construct the strain VHB63 [*trpC2 ΔrelQ relP::cmR rel:::spcR*] strain, first, two ≈2500 nucleotide-long DNA fragments were amplified by PCR using wt168 genomic DNA as a template: one located upstream of the *rel* ORF (primers: VHT29 and NOP13), another located downstream of the *rel* ORF (primers: NOP16 and VHT30). Second, spectinomycin resistant gene (*spcR*) of pDG1727 (Guerout-Fleury et al., 1995) was amplified by PCR using the primers NOP14 and NOP15. All three PCR products described above were used simultaneously as the template for PCR amplification using primers VHT29 and VHT30. The resulting long fragment was used to transform strain 168 [*trpC2*]. *rel* deletion mutants were selected for spectinomycin resistance, yielding the NHT437 [*trpC2 rel::spcR*] strain. Chromosomal DNA extracted from strain NHT437 was used to transform the strain NHT436 [*trpC2 ΔrelQ relP::cmR*] and selected for spectinomycin resistance yielding the VHB63 strain.

To construct the VHB47 strain [*trpC2 rplK::cmR*], first, two ≈1500 nucleotide-long DNA fragments were amplified by PCR using wt168 genomic DNA as a template: one located upstream of the *rplK* ORF (primers: VHT1 and VHT5), another located downstream of the *rplK* ORF (primers: VHT2 and VHT9). Second, the chloramphenicol-resistance marker (*cmR*) of pC194 (Horinouchi and Weisblum, 1982) was amplified by PCR using the primers VHT6 and VHT10. All three PCR products described above were used simultaneously as the template for PCR amplification using primers VHT1 and VHT2. The resulting long fragment was used to transform strain 168 (*trpC2*). *rplK* deletion mutants were selected by chloramphenicol resistance, yielding the strain VHB47. Chromosomal DNA extracted from strain VHB47 was used to transform the strain NBS1437 [*trpC2 relQ::tet relP::spcR*], and selected for chloramphenicol resistance, yielding the strain VHB49 [*trpC2 relQ::tet relP::spcR rplK::cmR*].

To construct the strain NBS1729 [*trpC2 rel-spcR*], First, two ≈1000 nt-long DNA fragments were amplified by PCR using genomic DNA as a template: one located +1452 ~ +2205 relative to the translation initiation codon of the rel *rel* ORF (primers: NOP1 and NOP2), another located downstream of the *rel* ORF (primers: NOP5 and NOP6). Second, the spectinomycin resistant gene (*spcR*) of pDG1727 (Guerout-Fleury et al., 1995) was amplified by PCR using the primers NOP3 and NOP4. All three PCR products described above were used simultaneously as the template for PCR amplification using primers NOP1 and NOP6. The resulting long fragment was used to transform strain 168 (*trpC2*). Transformants were selected for spectinomycin resistance yielding the strain NBS1729.

Strain VHB68 [*trpC2 relH420E-spcR*] was constructed using the site-directed mutagenesis technique (Heckman and Pease, 2007). To exchange the CAC codon (coding for Histidine) at position 420 for AGA (coding for Glutamic acid), two ≈2500 nucleotide-long overlapping DNA fragments were amplified by PCR using genomic DNA of strain NBS1729 as a template. The first fragment used the region located upstream of the *rel* ORF as the DNA template, spanning to codon 420 (primer, VHT29 and VHT32). The second used the region starting with the codon 420 as a template, spanning downstream of the *rel* ORF to include the *spcR* resistance marker (primers: VHT30 and VHT31). Two PCR products described above were used simultaneously as the template for PCR amplification using primers VHT29 and VHT30. The resulting long fragment was used to transform strain 168 (*trpC2*). The transformant was selected for spectinomycin resistance, yielding the strain VHB68. The desired mutation was confirmed by sequencing. Chromosomal DNA extracted from strain VHB68 was used to transform the strain NHT436, and bacteria were selected for spectinomycin resistance, yielding the strain VHB60 [*trpC2 ΔrelQ relP::cmR relH420E-spcR*].

Strain VHB144 [*trpC2 relC602C603-spcR*] was constructed using the site-directed mutagenesis technique. To exchange the TGC codon (coding for Cysteine) at position 602 and 603 for GCC (coding for Alanine), two ≈2500 nucleotide-long overlapping DNA fragments were amplified by PCR using the genomic DNA of strain NBS1729 strain as a template. The first fragment used the region located upstream of the *rel* ORF as the DNA template, spanning to codon 603 (primers: VHT29 and VHT40). The second used as a template the region starting with the codon 602 of *rel* ORF, spanning downstream of the *rel* ORF to include the *spcR* resistance marker (primers: VHT30 and VHT39). The two PCR products described above were used simultaneously as the template for PCR amplification using primers VHT29 and VHT30. The resulting long fragment was used to transform strain 168 (*trpC2*). The transformant was selected by spectinomycin resistance yielding the strain VHB144. The desired mutation was confirmed by sequencing. Chromosomal DNA extracted from strain VHB144 was used to transform the strain NHT436, and selected for spectinomycin resistance, yielding the strain VHB62 [*trpC2 ΔrelQ relP::cmR relC602AC603A-spcR*].

To construct the strain VHB155 [*trpC2 amyE::*$P_{hy\text{-}spank}$*-rel cmR rel::ermR*], the region containing *rel* ORF as well as the upstream Shine-Dalgarno sequence was amplified by PCR using *B. subtilis* 168 wild-type genomic DNA as a template (primers: NSP1 and NSP2). The PCR product was digested with SalI and SphI restriction enzymes and inserted into the SalI and SphI sites of plasmids pHT001 encoding i) a chloramphenicol-resistance marker (*cmR*), ii) a polylinker downstream of isopropyl-β-D-thiogalactopyranoside (IPTG) inducible $P_{hy\text{-}spank}$ promoter and iii) the lac repressor ORF (all three inserted in the middle of the *amyE* gene) (Takada et al., 2014), yielding the plasmid pHT101 (pHT001-*rel*, containing *rel* gene whose

transcription is controlled by $P_{hy\text{-}spank}$ promoter) which was used to transform wild type 168 strain. Selection for chloramphenicol resistance yielded the VHB145 [*trpC2 amyE::*$P_{hy\text{-}spank}$-*rel cmR*]. DNA extracted from strain RIK900 (*trpC2 rel::ermR*) was used to transform the strain VHB145, and selected by erythromycin resistance yielding the desired strain VHB155. To construct the strain VHB183 [*trpC2 relQ::tet relP::spcR amyE*::$P_{hy\text{-}spnak}$-*rel cmR rel::ermR*], plasmid pHT101 was used to transform strain NBS1437, and selected for chloramphenicol resistance, yielding the strain VHB175 [*trpC2 relQ::tet relP::spcR amyE*::$P_{hy\text{-}spnak}$-*rel cmR*]. DNA extracted from strain RIK900 was used to transform the strain VHB175, and selected for erythromycin resistance, yielding the desired strain VHB183.

To construct the strain VHB156 [*trpC2 amyE::*$P_{hy\text{-}spank}$-*relD264G cmR rel::ermR*], plasmid pHT101 was subjected to site-directed mutagenesis using primer NHP1 and NHP2 , according to directions of Phusion Site-Directed Mutagenesis Kit (Thermo Fisher Scientific), yielding the plasmid pHT125 (pHT001-*relD264G*) which was used to transform wt168 strain. Selection for chloramphenicol resistance yielded the strain VHB151 [*trpC2 amyE::*$P_{hy\text{-}spank}$-*relD264G cmR*]. DNA extracted from strain RIK900 was used to transform the strain VHB145, and selected for erythromycin resistance, yielding the desired strain VHB156. To construct the strain VHB187 [*trpC2 relQ::tet relP::spcR amyE*::$P_{hy\text{-}spnak}$-*relD264G cmR rel::ermR*], plasmid pHT125was used to transform the strain NBS1437, and selected for chloramphenicol resistance, yielding the strain VHB179 [*trpC2 relQ::tet relP::spcR amyE*::$P_{hy\text{-}spnak}$-*relD264G cmR*]. DNA extracted from strain RIK900 was used to transform the strain VHB179, and selected by erythromycin resistance, yielding the desired strain VHB187.

To construct the strain VHB159 [*trpC2 amyE::*$P_{hy\text{-}spank}$-*rel* $^{\Delta RRM}$ *cmR rel::ermR*], plasmid pHT101 was subjected to site-directed mutagenesis using primer VHT68 and VHT69 , according to directions of Phusion Site-Directed Mutagenesis Kit (Thermo Fisher Scientific), yielding the plasmid VHP435 (pHT001- *rel* $^{\Delta RRM}$), which was used to transform wild type 168 strain. Selection for chloramphenicol resistance yielded the strain VHB146 [*trpC2 amyE::*$P_{hy\text{-}spank}$-*rel* $^{\Delta RRM}$ *cmR*]. DNA extracted from strain RIK900was used to transform the strain VHB146, and selected by erythromycin resistance, yielding the desired strain VHB159. To construct the strain VHB184 [*trpC2 relQ::tet relP::spcR amyE*::$P_{hy\text{-}spnak}$-*rel* $^{\Delta RRM}$ *cmR rel::ermR*], plasmid VHP435 was used to transform the strain NBS1437, and selected by chloramphenicol resistance, yielding the strain VHB176 [*trpC2 relQ::tet relP::spcR amyE*::$P_{hy\text{-}spnak}$-*rel* $^{\Delta RRM}$ *cmR*]. The genomic DNA of the RIK900 strain was used to transform the strain VHB176, after selection for erythromycin resistance yielding the desired strain VHB184.

To construct the strain VHB160 [*trpC2 amyE::*$P_{hy\text{-}spank}$-*rel* $^{\Delta ZFD\text{-}RRM}$ *cmR rel::ermR*], plasmid pHT101 was subjected to site-directed mutagenesis using primer VHT69 and VHT189, according to directions of Phusion Site-Directed Mutagenesis Kit (Thermo Fisher Scientific), yielding VHP434 (pHT001- *rel* $^{\Delta ZFD\text{-}RRM}$) plasmid which was used to transform wt168 strain. Selection for chloramphenicol resistance yielded the strain VHB147 [*trpC2 amyE::*$P_{hy\text{-}spank}$-*rel* $^{\Delta ZFD\text{-}RRM}$ *cmR*]. The genomic DNA of the RIK900 strain was used to transform the strain VHB147, after selection for erythromycin resistance yielding the desired strain VHB160. To construct the strain VHB185 [*trpC2 relQ::tet relP::spcR amyE*::$P_{hy\text{-}spnak}$-*rel* $^{\Delta ZFD\text{-}RRM}$ *cmR rel::ermR*], plasmid VHP434 was used to transform strain NBS1437, and selected by chloramphenicol resistance, yielding the strain VHB177 [*trpC2 relQ::tet relP::spcR amyE*::$P_{hy\text{-}spnak}$-*rel* $^{\Delta ZFD\text{-}RRM}$ *cmR*]. The genomic DNA of the RIK900 strain was used to transform the strain VHB177, and selected by erythromycin resistance, yielding the desired strain VHB185.

To construct the strain VHB161 [*trpC2 amyE::*$P_{hy\text{-}spank}$*-rel(1-373)* $^{\Delta CTD}$ *cmR rel::ermR*], the pHT101 plasmid was subjected to site-directed mutagenesis using primers VHT69 and VHT190, as per manufacturers manual (Phusion Site-Directed Mutagenesis Kit, Thermo Fisher Scientific), yielding the plasmid VHP433 (pHT001-*rel(1-373)* $^{\Delta CTD}$) which was used to transform wt168 strain. Selection for chloramphenicol resistance yielded the strain VHB148 [*trpC2 amyE::*$P_{hy\text{-}spank}$*-rel(1-373)* $^{\Delta CTD}$ *cmR*]. DNA extracted from strain RIK900 was used to transform the strain VHB148, and selected by erythromycin resistance, yielding the desired strain VHB161. To construct the strain VHB186 [*trpC2 relQ::tet relP::spcR amyE*::$P_{hy\text{-}spnak}$-*rel(1-373)* $^{\Delta CTD}$ *cmR rel::ermR*], plasmid VHP433 was used to transform strain NBS1437, and selected by chloramphenicol resistance, yielding the strain VHB178 [*trpC2 relQ::tet relP::spcR amyE*::$P_{hy\text{-}spnak}$-*rel(1-373)* $^{\Delta CTD}$ *cmR*]. The genomic DNA of the RIK900 strain was used to transform the strain VHB178, and selected by erythromycin resistance, yielding the desired strain VHB186.

To construct the strain VHB162 [*trpC2 amyE::*$P_{hy\text{-}spank}$*-reD264G* $^{\Delta RRM}$ *cmR rel::ermR*], plasmid pHT125 was subjected to site-directed mutagenesis using primers VHT68 and VHT69, according to directions of Phusion Site-Directed Mutagenesis Kit (Thermo Fisher Scientific), yielding plasmid VHP438 (pHT001-*relD264G*$^{\Delta RRM}$) which was used to transform wt168 strain. Selection for chloramphenicol resistance yielded the strain VHB152 [*trpC2 amyE::*$P_{hy\text{-}spank}$*-relD264G* $^{\Delta RRM}$ *cmR*]. DNA extracted from strain RIK900 (*trpC2 rel::ermR*) was used to transform the strain VHB152, and selected by erythromycin resistance, yielding the desired strain VHB162. To construct the strain VHB188 [*trpC2 relQ::tet relP::spcR amyE*::$P_{hy\text{-}spnak}$-*relD264G* $^{\Delta RRM}$ *cmR rel::ermR R*], plasmid VHP438 was used to transform strain NBS1437, and selected by chloramphenicol resistance, yielding the strain VHB180 [*trpC2 relQ::tet relP::spcR amyE*::$P_{hy\text{-}spnak}$-*relD264G* $^{\Delta RRM}$ *cmR*]. The genomic DNA of the RIK900 strain was used to transform the strain VHB180, and selected by erythromycin resistance, yielding the desired strain VHB188.

To construct the strain VHB163 [*trpC2 amyE::*$P_{hy\text{-}spank}$*-relD264G* $^{\Delta ZFD\text{-}RRM}$ *cmR rel::ermR*], plasmid pHT125 was subjected to site-directed mutagenesis using primers VHT69 and VHT189, as per manufacturers manual (Phusion Site-Directed Mutagenesis Kit, Thermo Fisher Scientific), yielding the VHP437 plasmid (pHT001-*relD264G* $^{\Delta ZFD\text{-}RRM}$) which was used to transform wt168 strain. Selection for chloramphenicol resistance yielded the strain VHB153 [*trpC2 amyE::*$P_{hy\text{-}spank}$*-relD264G* $^{\Delta ZFD\text{-}RRM}$ *cmR*]. DNA extracted from strain RIK900 was used to transform the strain VHB153, and selected by erythromycin resistance, yielding the desired strain VHB163. To construct the strain VHB189 [*trpC2 relQ::tet relP::spcR amyE*::$P_{hy\text{-}spnak}$-*relD264G* $^{\Delta ZFD\text{-}RRM}$ *cmR rel::ermR R*], plasmid VHP437 was used to transform strain NBS1437, and selected by chloramphenicol resistance, yielding the strain VHB181 [*trpC2 relQ::tet relP::spcR amyE*::$P_{hy\text{-}spnak}$-*relD264G* $^{\Delta ZFD\text{-}RRM}$ *cmR*]. The genomic DNA of the RIK900 strain was used to transform the strain VHB181, and selected by erythromycin resistance, yielding the desired strain VHB189.

To construct the strain VHB161 [*trpC2 amyE::*$P_{hy\text{-}spank}$*-relD264G(1-373)* $^{\Delta CTD}$ *cmR rel::ermR*], plasmid pHT125 was subjected to site-directed mutagenesis using primer VHT69 and VHT190 , according to directions of Phusion Site-Directed Mutagenesis Kit (Thermo Fisher Scientific), yielding plasmid VHP436 (pHT001-*relD264G* $^{\Delta CTD}$) which was used to transform wt168 strain. Selection for chloramphenicol resistance yielded the strain VHB154 [*trpC2 amyE::*$P_{hy\text{-}spank}$*-relD264G* $^{\Delta CTD}$ *cmR*]. DNA extracted from strain RIK900 was used to transform the strain VHB154, and selected by erythromycin resistance, yielding the desired strain VHB164. To construct the strain VHB190 [*trpC2 relQ::tet relP::spcR amyE*::$P_{hy\text{-}spnak}$-*relD264G* $^{\Delta CTD}$ *cmR rel::ermR*], plasmid VHP436 was used to transform strain NBS1437

[*trpC2 relQ::tet relP::spcR*], and selected by chloramphenicol resistance, yielding the strain VHB182 [*trpC2 relQ::tet relP::spcR amyE*::$P_{hy\text{-}spnak}$-*relD264G* $^{\Delta CTD}$ *cmR*]. The genomic DNA of the RIK900 strain was used to transform the strain VHB182, and selected by erythromycin resistance, yielding the desired strain VHB190.

To construct the strain VHB231 [*trpC2 relQ::tet relP::spcR amyE*::$P_{hy\text{-}spnak}$-*relH420E* $^{\Delta RRM}$ *cmR rel::ermR*], plasmid VHP435 was subjected to site-directed mutagenesis using primer VHT337 and VHT338 , according to directions of Phusion Site-Directed Mutagenesis Kit (Thermo Fisher Scientific), yielding plasmid VHP591 (pHT001-*relH420E* $^{-\Delta RRM}$ *amp cmR*) which was used to transform strain NBS1437. Selection for chloramphenicol resistance yielded the strain VHB230 [*trpC2 relQ::tet relP::spcR amyE*::$P_{hy\text{-}spnak}$-*relH420E* $^{\Delta RRM}$ *cmR*]. DNA extracted from strain RIK900 (*trpC2 rel::ermR*) was used to transform the strain VHB230, and selected by erythromycin resistance, yielding the desired strain VHB231.

To construct the strain VHB233 [*trpC2 relQ::tet relP::spcR amyE*::$P_{hy\text{-}spnak}$-*relC602AC603A* $^{\Delta RRM}$ *cmR rel::ermR*], VHP435 was subjected to site-directed mutagenesis using primer VHT337 and VHT338 , according to directions of Phusion Site-Directed Mutagenesis Kit (Thermo Fisher Scientific), yielding plasmid VHP592 (pHT001-*relC602AC603A* $^{-\Delta RRM}$ *amp cmR*) which was used to transform strain NBS1437. Selection for chloramphenicol resistance yielded the VHB232 [*trpC2 relQ::tet relP::spcR amyE*::$P_{hy\text{-}spnak}$-*relC602AC603A* $^{\Delta RRM}$ *cmR*]. DNA extracted from strain RIK900 was used to transform the strain VHB232, and selected by erythromycin resistance, yielding the desired strain VHB233.

To construct the strain VHB252 [*trpC2 amyE::*$P_{hy\text{-}spank}$-*rel(1-155)* $^{\Delta CTD}$ *cmR rel::ermR*], the pHT101 plasmid was subjected to site-directed mutagenesis using primers VHT69 and VHT341, as per manufacturers manual (Phusion Site-Directed Mutagenesis Kit, Thermo Fisher Scientific), yielding the plasmid VHP622 (pHT001-*rel(1-155)* $^{\Delta CTD}$) which was used to transform wt168 strain. Selection for chloramphenicol resistance yielded the strain VHB249 [*trpC2 amyE::*$P_{hy\text{-}spank}$-*rel(1-155)* $^{\Delta CTD}$ *cmR*]. DNA extracted from strain RIK900 was used to transform the strain VHB249, and selected by erythromycin resistance, yielding the desired strain VHB252.

To construct the strain VHB253 [*trpC2 amyE::*$P_{hy\text{-}spank}$-*rel(1-196)* $^{\Delta CTD}$ *cmR rel::ermR*], the pHT101 plasmid was subjected to site-directed mutagenesis using primers VHT69 and VHT342, as per manufacturers manual (Phusion Site-Directed Mutagenesis Kit, Thermo Fisher Scientific), yielding the plasmid VHP623 (pHT001-*rel(1-196)* $^{\Delta CTD}$) which was used to transform wt168 strain. Selection for chloramphenicol resistance yielded the strain VHB250 [*trpC2 amyE::*$P_{hy\text{-}spank}$-*rel(1-196)* $^{\Delta CTD}$ *cmR*]. DNA extracted from strain RIK900 was used to transform the strain VHB250, and selected by erythromycin resistance, yielding the desired strain VHB253.

To construct the strain VHB254 [*trpC2 amyE::*$P_{hy\text{-}spank}$-*rel(1-336)* $^{\Delta CTD}$ *cmR rel::ermR*], the pHT101 plasmid was subjected to site-directed mutagenesis using primers VHT69 and VHT344, as per manufacturers manual (Phusion Site-Directed Mutagenesis Kit, Thermo Fisher Scientific), yielding the plasmid VHP624 (pHT001-*rel(1-336)* $^{\Delta CTD}$) which was used to transform wt168 strain. Selection for chloramphenicol resistance yielded the strain VHB251 [*trpC2 amyE::*$P_{hy\text{-}spank}$-*rel(1-336)* $^{\Delta CTD}$ *cmR*]. DNA extracted from strain RIK900 was used to transform the strain VHB251, and selected by erythromycin resistance, yielding the desired strain VHB254.

To construct the strain VHB281 [*trpC2 relQ::tet relP::spcR amyE*::$P_{hy\text{-}spnak}$-*relR619E* $^{\Delta RRM}$ *cmR rel::ermR*], plasmid VHP435 was subjected to site-directed mutagenesis using primer

VHT413 and VHT414 , according to directions of Phusion Site-Directed Mutagenesis Kit (Thermo Fisher Scientific), yielding plasmid VHP626 (pHT001-*relR619E* $^{\Delta RRM}$) which was used to transform strain NBS1437. Selection for chloramphenicol resistance yielded the strain VHB280 [*trpC2 relQ::tet relP::spcR amyE*::$P_{hy\text{-}spnak}$-*relR619E* $^{\Delta RRM}$ *cmR*]. DNA extracted from strain RIK900 (*trpC2 rel::ermR*) was used to transform the strain VHB, and selected by erythromycin resistance, yielding the desired strain VHB281.

To construct the strain VHB282 [*trpC2 amyE::*$P_{hy\text{-}spank}$-*relR619E cmR rel::ermR*], plasmid pHT101 was subjected to site-directed mutagenesis using primer VHT413 and VHT414 , according to directions of Phusion Site-Directed Mutagenesis Kit (Thermo Fisher Scientific), yielding the plasmid VHP625 (pHT001-*relR619E*) which was used to transform wt168 strain. Selection for chloramphenicol resistance yielded the strain VHB273 [*trpC2 amyE::*$P_{hy\text{-}spank}$-*relR619E cmR*]. DNA extracted from strain RIK900 was used to transform the strain VHB273, and selected for erythromycin resistance, yielding the desired strain VHB282.

Strain VHB455 [*trpC2 relN685A-spcR*] was constructed using the site-directed mutagenesis technique. To exchange the AAT codon (coding for Asparagine) at position 685 for GCG (coding for Alanine), two ≈2,500 nucleotide-long overlapping DNA fragments were amplified by PCR using genomic DNA of strain NBS1729 strain as a template. The first fragment used as the DNA template the region located upstream of the *rel* ORF, spanning to codon 685 of the *rel* ORF (primers: VHT29 and VHT83). The second used as a template the region starting with the codon 685 of *rel* ORF, spanning downstream of the *rel* ORF to include the *spcR* resistance marker (primers: VHT30 and VHT82). The two PCR products described above were used simultaneously as the template for PCR amplification using primers VHT29 and VHT30. The resulting long fragment was used to transform wild-type strain 168 (*trpC2*). The transformants were selected by spectinomycin resistance yielding the strain VHB455. The desired mutation was confirmed by sequencing.

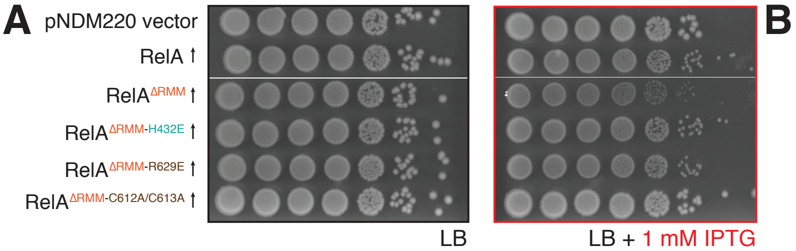

Supplementary Figure S1. The toxicity of ΔRRM *E. coli* RelA expressed in the Δ*relA* background is mitigated by mutations compromising interactions with tRNA and the ribosome. *E. coli* BW25113 Δ*relA* cells were transformed with low copy IPTG-inducible vector pNDM220 (vector) or pNDM220-based constructs expressing either wild-type and mutant versions of *E. coli* RelA as indicated on the figure. Ten-fold serial dilutions of overnight LB cultures were made and spotted onto LB agar (LB) supplemented with 30 μg/mL ampicillin, without (**A**, left panel) and with 1 mM IPTG (**B**, right panel). The plates were incubated at 37 °C and scored after 18 hours. Growth assays presented on panels **A** and **B** originate from the same individual plates after cropping and splicing together.

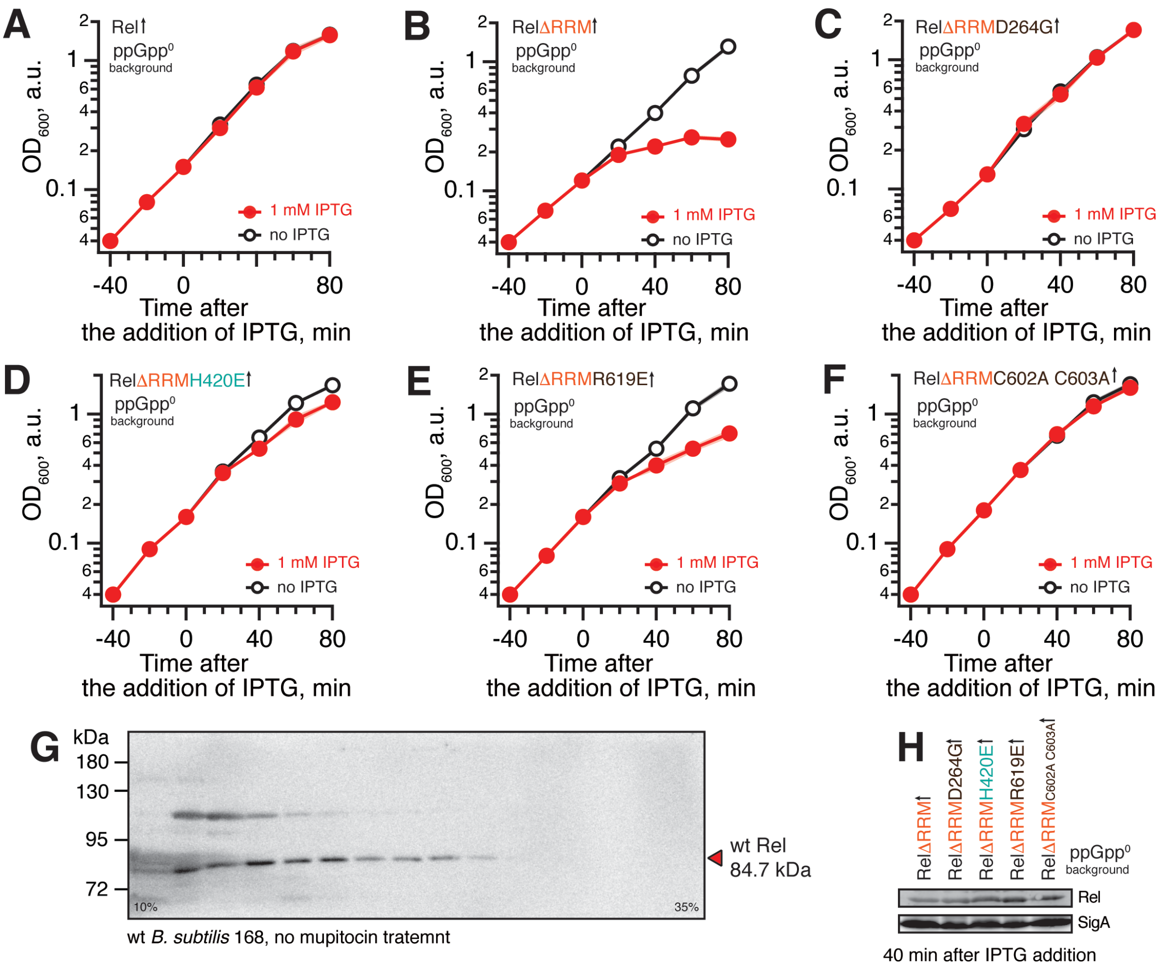

Supplementary Figure S2. The toxicity of *B. subtilis* ΔRRM Rel is mediated by (p)ppGpp synthesis and is countered by mutations compromising the interaction with starved ribosomes. Full-length (**A**: VHB155) or C-terminally ΔRRM truncated Rel variants (**B**: VHB184; **C**: VHB188; **D**: VHB231; **E**: VHB281; **F**: VHB233) were expressed in ppGpp^0 *B. subtilis* growing in liquid LB medium at 37 °C. The error bars represent standard deviation of the mean (n = 3) and are shown as shading since they are generally smaller than the markers. (**G**) Uncut version of the immunoblot analysis presented on **Figure 4A**, top line. (**H**) anti-Rel and anti-SigA immunoblot analyses of samples collected 40 minutes upon induction of expression Rel$^{\Delta RRM}$ or D264G, H420E, R619E and C602A C603A Rel$^{\Delta RRM}$ variants by IPTG.

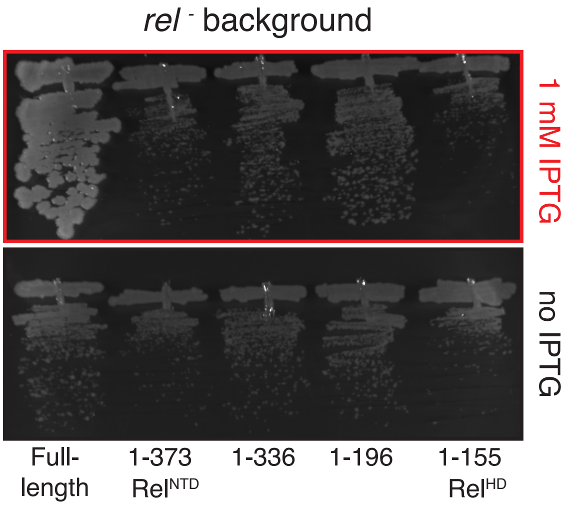

Supplementary Figure S3. None of the tested truncated variants of Rel^{NTD} rescue the growth effect of Δ*rel B. subtilis*. Either full-length or N-terminally truncated Rel variants were expressed in Δ*rel B. subtilis* (VHB155, VHB161, VHB252-254) growing on solid LB medium at 37 °C and plates were scored after 18 hour incubation.

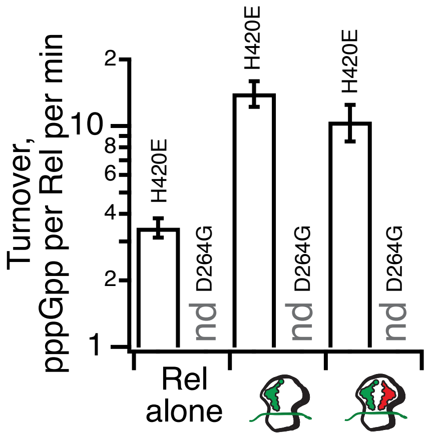

Supplementary Figure S4. ***B. subtilis*** **D264G Rel is catalytically inactive and** ***B. subtilis*** **H420E Rel is not activated by A-site deacylated tRNA.** ^{3}H-pppGpp synthesis activity of 140 nM of Rel H420E and Rel D264G assayed in the presence of 1 mM ATP nd 0.3 mM of either ^{3}H-labelled GTP. As indicated on the figure, the reaction mixtures were supplemented with combinations of 0.5 µM IC (MV) and native *E. coli* $tRNA^{Val}$ (2 µM: A-site) as well as 100 µM pppGpp. All experiments were performed in HEPES:Polymix buffer, pH 7.5 at 37 °C in the presence of 5 mM Mg^{2+}. Error bars represent SDs of the turnover estimates by linear regression using four data points.

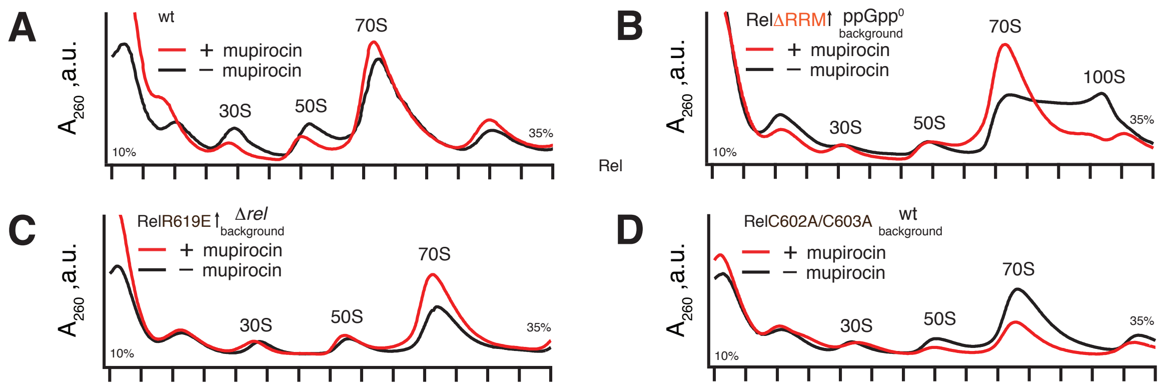

Supplementary Figure S5. Polysome profiles of Rel variants expressed either ectopically under the control of IPTG-inducible $P_{hy\text{-}spnak}$ promotor [ΔRRM Rel in ppGpp^{0} *B. subtilis* (VHB184), R619E Rel in Δ*rel B. subtilis* (VHB282)] or from the native chromosomal locus [C602A C603A mutant (VHB144)]. Expression of Rel$^{\Delta RRM}$ was induced by 1 mM IPTG for 10 minutes followed by a 10 minute challenge with 700 nM mupirocin. To drive the expression of RelR619E Rel the strain as grown in LB supplemented with 1 mM IPTG. In the case of R619E and C602AC603A Rel the culture was treated with mupirocin for 20 minutes.

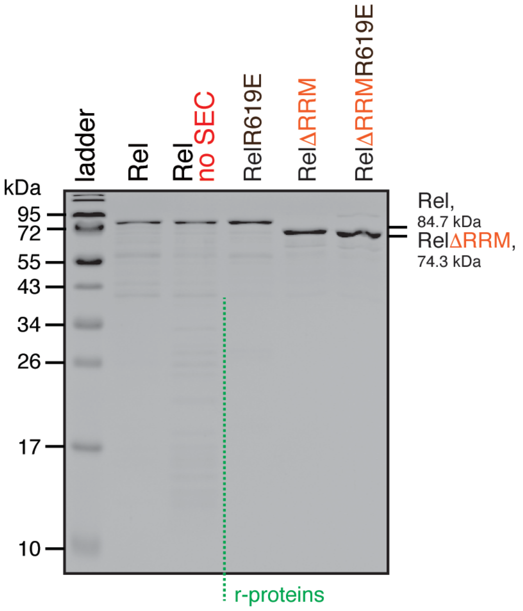

Supplementary Figure S6. SDS-PAGE analysis of wild-type Rel as well as ΔRRM, R619E and ΔRRM R619E mutant variants. The full-length Rel protein was purified either as described on **Figure 5** or with the SEC step omitted (no SEC).

Supplementary Table 1. ***E. coli*** **strains and plasmids used in this study.**

Strain / plasmid	Description	Reference
BL21 DE	BF⁻ *ompT gal dcm lon hsdS*$_B$(*r*$_B$⁻ *m*$_B$⁻) λ(DE3 [*lacI lacUV5-T7p07 ind1 sam7 nin5*]) [*malB*$^+$]$_{K-12}$(λ^S)	laboratory collection
BW25113	wild type *E. coli* K-12	laboratory collection
BW25113 **Δ*relA***	Δ*relA E. coli* BW25113	(Varik et al., 2016)
pNDM220	mini-R1 *lacI*^{q} P$_{A1/O4/O3}$ *amp*^{R}	(Gotfredsen and Gerdes, 1998)
pNDM220::*relA*	pNDM220::*relA* full-length (residues 1-744)	(Turnbull et al., 2019)
pNDM220::*relA*^{ΔRRM}	pNDM220::*relA* ΔRRM domain (residues 1-657)	(Turnbull et al., 2019)
pNDM220::*relA*$^{ΔRRM-H432E}$	pNDM220::*relA* ΔRRM, H432E mutant	this work
pNDM220::*relA*$^{ΔRRM-R629E}$	pNDM220::*relA* ΔRRM, R629E mutant	this work
pNDM220::*relA*$^{ΔRRM-C612A/C613A}$	pNDM220::*relA* ΔRRM, C612A/C613A mutant	this work
pMG25	pUC *lacI*^{q} P$_{A1/O4/O3}$ *amp*^{R}	(Turnbull et al., 2019)
pMG25::*relA*	pMG25::*relA* full-length (residues 1-744)	(Turnbull et al., 2019)
pMG25::*relA*^{ΔRRM}	pMG25::*relA* ΔRMM domain (residues 1-657)	this work
pMG25::*spoT*	pMG25::*spoT* full-length (residues 1-702)	this work
pMG25::*spoT*^{ΔRRM}	pMG25:: *spoT* ΔRMM domain (residues 1-617)	this work
pET24::*relA*	N-terminal His$_{10}$-SUMO::*relA*	(Turnbull et al., 2019)
pET24::*relA*^{H432E}	N-terminal His$_{10}$-SUMO::*relA*^{H432E}	this work

Supplementary Table 2. *B. subtilis* strains used in this study.

Strain	Description	Reference
wild type 168	*trpC2*	laboratory stock
VHB47	*trpC2 rplK::cmR*	this work
VHB49	*trpC2 relQ::tet relP::spcR rplK::cmR*	this work
VHB60	*trpC2ΔrelQ relP::cmR relH420E-spcR*	this work
VHB62	*trpC2 ΔrelQ relP::cmR relC602AC603A-spcR*	this work
VHB63	*trpC2 ΔrelQ relP::cmR rel:::spcR*	this work
VHB68	*trpC2 relH420E-spcR*	this work
VHB144	*trpC2 relC602AC603A-spcR*	this work
VHB145	*trpC2 amyE*::$P_{hy\text{-}spnak}$-*rel cmR*	this work
VHB146	*trpC2 amyE*::$P_{hy\text{-}spnak}$-$rel^{\Delta RRM}$ *cmR*	this work
VHB147	*trpC2 amyE*::$P_{hy\text{-}spnak}$-$rel^{\Delta ZFD\text{-}RRM}$ *cmR*	this work
VHB148	*trpC2 amyE*::$P_{hy\text{-}spnak}$-$rel(1\text{-}373)^{NTD}$ *cmR*	this work
VHB151	*trpC2 amyE*::$P_{hy\text{-}spnak}$-*relD264G cmR*	this work
VHB152	*trpC2 amyE*::$P_{hy\text{-}spnak}$-$relD264G^{\Delta RRM}$ *cmR*	this work
VHB153	*trpC2 amyE*::$P_{hy\text{-}spnak}$-$relD264G^{\Delta ZFD\text{-}RRM}$ *cmR*	this work
VHB154	*trpC2 amyE*::$P_{hy\text{-}spnak}$-$relD264G(1\text{-}373)^{NTD}$ *cmR*	this work
VHB155	*trpC2 amyE*::$P_{hy\text{-}spnak}$-*rel cmR rel::ermR*	this work
VHB156	*trpC2 amyE*::$P_{hy\text{-}spnak}$-*relD264G cmR rel::ermR*	this work
VHB159	*trpC2 amyE*::$P_{hy\text{-}spnak}$-$rel^{\Delta RRM}$ *cmR rel::ermR*	this work
VHB160	*trpC2 amyE*::$P_{hy\text{-}spnak}$-$rel^{\Delta ZFD\text{-}RRM}$ *cmR rel::ermR*	this work
VHB161	*trpC2 amyE*::$P_{hy\text{-}spnak}$-rel^{NTD} *cmR rel::ermR*	this work
VHB162	*trpC2 amyE*::$P_{hy\text{-}spnak}$-*relD264G* $^{\Delta RRM}$ *cmR rel::ermR*	this work
VHB163	*trpC2 amyE*::$P_{hy\text{-}spnak}$-$relD264G^{\Delta ZFD\text{-}RRM}$ *cmR rel::ermR*	this work
VHB164	*trpC2 amyE*::$P_{hy\text{-}spnak}$-$relD264G(1\text{-}373)^{NTD}$ *cmR rel::ermR*	this work
VHB175	*trpC2 relQ::tet relP::spcR amyE*::$P_{hy\text{-}spnak}$-*rel cmR*	this work
VHB176	*trpC2 relQ::tet relP::spcR amyE*::$P_{hy\text{-}spnak}$-$rel^{\Delta RRM}$ *cmR*	this work
VHB177	*trpC2 relQ::tet relP::spcR amyE*::$P_{hy\text{-}spnak}$-$rel^{\Delta ZFD\text{-}RRM}$ *cmR*	this work
VHB178	*trpC2 relQ::tet relP::spcR amyE*::$P_{hy\text{-}spnak}$-$rel(1\text{-}373)^{NTD}$ *cmR*	this work
VHB179	*trpC2 relQ::tet relP::spcR amyE*::$P_{hy\text{-}spnak}$-*relD264G cmR*	this work
VHB180	*trpC2 relQ::tet relP::spcR amyE*::$P_{hy\text{-}spnak}$-$relD264G^{\Delta RRM}$ *cmR*	this work
VHB181	*trpC2 relQ::tet relP::spcR amyE*::$P_{hy\text{-}spnak}$-$relD264G^{\Delta ZFD\text{-}RRM}$ *cmR*	this work
VHB182	*trpC2 relQ::tet relP::spcR amyE*::$P_{hy\text{-}spnak}$-$relD264G(1\text{-}373)^{NTD}$ *cmR*	this work
VHB183	*trpC2 relQ::tet relP::spcR amyE*::$P_{hy\text{-}spnak}$-*rel cmR rel::ermR*	this work
VHB184	*trpC2 relQ::tet relP::spcR amyE*::$P_{hy\text{-}spnak}$-$rel^{\Delta RRM}$ *cmR rel::ermR*	this work
VHB185	*trpC2 relQ::tet relP::spcR amyE*::$P_{hy\text{-}spnak}$-$rel^{\Delta ZFD\text{-}RRM}$	this work

	cmR rel::ermR	
VHB186	*trpC2 relQ::tet relP::spcR amyE*::P$_{hy\text{-}spnak}$-*rel(1-373)*^{NTD} *cmR rel::ermR*	this work
VHB187	*trpC2 relQ::tet relP::spcR amyE*::P$_{hy\text{-}spnak}$-*relD264G cmR rel::ermR*	this work
VHB188	*trpC2 relQ::tet relP::spcR amyE*::P$_{hy\text{-}spnak}$-*relD264G*$^{\Delta RRM}$ *cmR rel::ermR*	this work
VHB189	*trpC2 relQ::tet relP::spcR amyE*::P$_{hy\text{-}spnak}$-*relD264G*$^{\Delta ZFD\text{-}RRM}$ *cmR rel::ermR*	this work
VHB190	*trpC2 relQ::tet relP::spcR amyE*::P$_{hy\text{-}spnak}$-*relD264G(1-373)*^{NTD} *cmR rel::ermR*	this work
VHB230	*trpC2 relQ::tet relP::spcR amyE*::P$_{hy\text{-}spnak}$-*relH420E*$^{\Delta RRM}$ *cmR*	this work
VHB231	*trpC2 relQ::tet relP::spcR amyE*::P$_{hy\text{-}spnak}$-*relH420E*$^{\Delta RRM}$ *cmR rel::ermR*	this work
VHB232	*trpC2 relQ::tet relP::spcR amyE*::P$_{hy\text{-}spnak}$-*relC602AC603A*$^{\Delta RRM}$ *cmR*	this work
VHB233	*trpC2 relQ::tet relP::spcR amyE*::P$_{hy\text{-}spnak}$-*relC602AC603A*$^{\Delta RRM}$ *cmR rel::ermR*	this work
VHB249	*trpC2 amyE*::P$_{hy\text{-}spnak}$-*rel(1-155)*^{NTD} *cmR*	this work
VHB250	*trpC2 amyE*::P$_{hy\text{-}spnak}$-*rel(1-196)*^{NTD} *cmR*	this work
VHB251	*trpC2 amyE*::P$_{hy\text{-}spnak}$-*rel(1-336)*^{NTD} *cmR*	this work
VHB252	*trpC2 amyE*::P$_{hy\text{-}spnak}$-*rel(1-155)*^{NTD} *cmR rel::ermR*	this work
VHB253	*trpC2 amyE*::P$_{hy\text{-}spnak}$-*rel(1-196)*^{NTD} *cmR rel::ermR*	this work
VHB254	*trpC2 amyE*::P$_{hy\text{-}spnak}$-*rel(1-336)*^{NTD} *cmR rel::ermR*	this work
VHB273	*trpC2 amyE*::P$_{hy\text{-}spnak}$-*relR619E cmR*	this work
VHB280	*trpC2 relQ::tet relP::spcR trpC2 amyE*::P$_{hy\text{-}spnak}$-*relR619E*$^{\Delta RRM}$ *cmR*	this work
VHB281	*trpC2 relQ::tet relP::spcR trpC2 amyE*::P$_{hy\text{-}spnak}$-*relR619E*$^{\Delta RRM}$ *cmR rel::ermR*	this work
VHB282	*trpC2 amyE*::P$_{hy\text{-}spnak}$-*relR619E cmR rel::ermR*	this work
VHB455	*trpC2 relN685A-spcR*	this work
RIK900	*trpC2 rel::ermR*	(Nanamiya et al., 2008)
RIK908	*trpC2 relP::spcR*	(Nanamiya et al., 2008)
RIK1000	*trpC2 ΔrelQ*	(Nanamiya et al., 2008)
RIK1004	*trpC2 relQ::cmR*	(Tagami et al., 2012)
RIK2508	*trpC2 Δhpf*	(Akanuma et al., 2016)
NBS1393	*trpC2 relQ::tet*	this work
NBS1437	*trpC2 relQ::tet relP::spcR*	this work
NBS1729	*trpC2 rel-spcR*	this work
NHT436	*trpC2 ΔrelQ relP::cmR*	this work
NHT437	*trpC2 rel::spcR*	this work

Supplementary Table 3. ***B. subtilis*** **plasmids used in this study.**

Plasmid	Description	Reference
pHT101	pHT001-*rel amp cmR*	this work
pHT125	pHT001-*relD264G amp cmR*	this work
VHP186	pET24d-*His*$_{10}$-*SUMO-rel kmR*	this work
VHP187	pET24d-*His*$_{10}$-*SUMO-relD264G kmR*	this work
VHP230	pET24d-*His*$_{10}$-*SUMO-relH420E kmR*	this work
VHP231	pET24d-*His*$_{10}$-*SUMO-rel*$^{\Delta RRM}$ *kmR*	this work
VHP433	pHT001-*rel(1-373)*^{NTD} *amp cmR*	this work
VHP434	pHT001-*rel*$^{\Delta ZFD\text{-}RRM}$ *amp cmR*	this work
VHP435	pHT001-*rel*$^{\Delta RRM}$ *amp cmR*	this work
VHP436	pHT001-*relD264G(1-373)* ^{NTD} *amp cmR*	this work
VHP437	pHT001-*relD264G*$^{\Delta ZFD\text{-}RRM}$ *amp cmR*	this work
VHP438	pHT001-*relD264G*$^{\Delta RRM}$ *amp cmR*	this work
VHP591	pHT001-*relH420E*$^{\Delta RRM}$ *amp cmR*	this work
VHP592	pHT001-*relC602AC603A*$^{\Delta RRM}$ *amp cmR*	this work
VHP622	pHT001-*rel(1-155)*^{NTD} *amp cmR*	this work
VHP623	pHT001-*rel(1-196)*^{NTD} *amp cmR*	this work
VHP624	pHT001-*rel(1-336)*^{NTD} *amp cmR*	this work
VHP625	pHT001-*relR619E amp cmR*	this work
VHP626	pHT001-*relR619E*$^{\Delta RRM}$ *amp cmR*	this work
VHP627	pET24d-*His*$_{10}$-*SUMO-relR619E kmR*	this work
VHP630	pET24d-*His*$_{10}$-*SUMO-relR619E*$^{\Delta RRM}$ *kmR*	this work

Supplementary Table 4. Primers used in this work.

Primer	Sequence, 5'-3'
VHT1	ATTAAGTGATTTAGAGAGAAAAGTACTCGTCTTATATCTCG
VHT2	AAGAGCAAGGTTGCGAACTGGA
VHT5	ATCTAAAGTGTTAGCTGGGTTAGCTTTTCCAGCAG
VHT6	CTAACCCAGCTAACACTTTAGATAAAAATTTAGGAGGC
VHT9	GCTTTTATAACGCAGTATGGGTATTGTAATCGAGGATTAA
VHT10	CCATACTGCGTTATAAAAGCCAGTCATTAGGCC
VHT29	AAGATCAAGCAAAGGCGTTTGACCTCGAT
VHT30	GTGCGTCAACGACGGGCACAGTC
VHT31	TTACCGGATTGAGTCTGAAATCGGCAATAAAACAATCGGT
VHT32	CGATTTCAGACTCAATCCGGTAAGAAAAGTCAATCGGAAC
VHT39	GTTTATCAAAAGCCGCCAATCCTGTGCCAGGTGATGATATTGTC
VHT40	GGCACAGGATTGGCGGCTTTTGATAAACGGACAAGGAGGTTGTCAAT
VHT68	CCACTCTACCGGGATCAGCCGCT
VHT69	TAAGCATGCAAGCTAATTCGGTGGAAACG
VHT82	GAAAACCGCGATTTCATCTGTCTCTGGCAAATCGGATCG
VHT83	CAGATGAAATCGCGGTTTTCGTTTCATTCACTGCCTGGAG
VHT123	CATTATCGCTCTCTCCTTCGTCGACTAAGCTAATTG
VHT125	TAAGCATGCAAGCTAATTCGGTGGAAACGAGG
VHT131	GGCGGCCATCATCATCATCATCACGCCAA
VHT169	CGACGAAGGAGAGAGCGATAATGGCGAACGAACAAGTATTG
VHT170	TGATGATGATGATGGCCGCCGTTCATGACGCGGCGCA
VHT189	CCGTTTTCTTCCTTGCGGGTATGGCT
VHT190	ATTTTGAAATTCTAAAATTTCACGGAACCAAGAAAGCTTTTTCTCAAAG
VHT337	CTCAATCCGGTAAGAAAAGTCAATCGGAACAGAACCGGA
VHT338	TCTGAAATCGGCAATAAAACAATCGGTGCCAAAGT
VHT339	GCCAATCCTGTGCCAGGTGATGATATTGTCGGCT

VHT340	GGCTTTTGATAAACGGACAAGGAGGTTGTCAATGCCCT
VHT341	CAGATGTTTCAGTGTCCGCATATTGTGAAGACGAT
VHT342	GTTCAAATAACGGAGCGCCGTATCTTCCAATTCCCA
VHT344	CCGCTATTTCATGCATTTCAAAGGTGCGGA
VHT383	CGACGAAGGAGAGAGCGATAATGGTTGCGGTAAGAAGTGCA
VHT384	CACCGAATTAGCTTGCATGCTCAACTCCCGTGCAACCGA
VHT385	CACCGAATTAGCTTGCATGCTCACCATACCGCGTCAACAAT
VHT386	CGACGAAGGAGAGAGCGATAATGCAAGTTATTGACTCTAAACT
VHT387	CACCGAATTAGCTTGCATGCTTAGCTGACCTCTTCATTATCATCCCA
VHT413	GAAGGGGTTTCGGTCCATCGCGAAGACTGT
VHT414	GCCTTTTGTGATAAAGCCGACAATATCATCACCTG
NOP1	GCGGCGTGAAGAAAATGTCG
NOP2	CACCTCGTTGTTAGTTCATGACGCGGCGCA
NOP3	CGTCATGAACTAACAACGAGGTGAAATCATGAG
NOP4	TCTAACCCCTTTACTAGGCCTAATTGAGAGAAG
NOP5	CAATTAGGCCTAGTAAAGGGGTTAGAAAAGAGATTAG
NOP6	CAGTCCTGCCAAAGATCAGGAAC
NOP7	ATTGAACTGGCAGGCCT
NOP8	TACCTCTGAAAGCTGCTGG
NOP9	GGGATGTATGTAGCGGTTATGAAGTGAAATTGA
NOP10	TCCCCTTTACTTACTAGAAATCCCTTTGAGAATG
NOP11	AGGGATTTCTAGTAAGTAAAGGGGAAGAAGAGCATG
NOP12	TTTCGGATGCAACTTGGC
NOP13	CTCGTTGTTATGCTCGGCAGTCAATACTTGTTCGT
NOP14	CTGCCGAGCATAACAACGAGGTGAAATCATGAG
NOP15	AGAATAGATATTACTAGGCCTAATTGAGAGAAG
NOP16	GGCCTAGTAATATCTATTCTGTGCGCCGCGTCA
NSP1	ACATGCATGCTTAGTTCATGACGCGGC

NSP2	ACGCGTCGACCTTGATAGTAAACTACAATACAGTTTACA
NHP1	GGCTTGTTGGCTGTCCGTATTCTTGTGAATAGC
NHP2	GTAAATTTCATTGAATTGCTTATTTTGCAGCACCATTTTTCG
pQErelA_F	GCCCCATGGATGGCGAACGAACAAGTATTG
pQErelA_R	GTCCCATGGGTTCATGACGCGGCGCACAG
MR1	CCCCCGGATCCTGGTCCCTAAAGGAGAGG
MR2	CCCCCCTCGAGTTACCATACCGCGTCAACAATGC
MR3	GGACTTCGCTTACCACATCGAGAGTGATGTCGGACACCGCTG
MR4	CAGCGGTGTCCGACATCACTCTCGATGTGGTAAGCGAAGTCC
MR5	GCTTCATTACCCAGGGGGAGGGTATTTCAGTACACCG
MR6	CGGTGTACTGAAATACCCTCCCCCTGGGTAATGAAGC
MR7	GCACCACATCGCGCGCGCCGCCCAGCCGATTCCTGGAG
MR8	CTCCAGGAATCGGCTGGGCGGCGCGCGCGATGTGGTGC

Supplementary references:

Akanuma, G., Kazo, Y., Tagami, K., Hiraoka, H., Yano, K., Suzuki, S., Hanai, R., Nanamiya, H., Kato-Yamada, Y., and Kawamura, F. (2016). Ribosome dimerization is essential for the efficient regrowth of *Bacillus subtilis*. *Microbiology* 162**,** 448-458.

Gotfredsen, M., and Gerdes, K. (1998). The *Escherichia coli relBE* genes belong to a new toxin-antitoxin gene family. *Mol Microbiol* 29**,** 1065-1076.

Guerout-Fleury, A.M., Shazand, K., Frandsen, N., and Stragier, P. (1995). Antibiotic-resistance cassettes for *Bacillus subtilis*. *Gene* 167**,** 335-336.

Heckman, K.L., and Pease, L.R. (2007). Gene splicing and mutagenesis by PCR-driven overlap extension. *Nat Protoc* 2**,** 924-932.

Ho, S.N., Hunt, H.D., Horton, R.M., Pullen, J.K., and Pease, L.R. (1989). Site-directed mutagenesis by overlap extension using the polymerase chain reaction. *Gene* 77**,** 51-59.

Horinouchi, S., and Weisblum, B. (1982). Nucleotide sequence and functional map of pC194, a plasmid that specifies inducible chloramphenicol resistance. *J Bacteriol* 150**,** 815-825.

Nanamiya, H., Kasai, K., Nozawa, A., Yun, C.S., Narisawa, T., Murakami, K., Natori, Y., Kawamura, F., and Tozawa, Y. (2008). Identification and functional analysis of novel (p)ppGpp synthetase genes in *Bacillus subtilis*. *Mol Microbiol* 67**,** 291-304.

Tagami, K., Nanamiya, H., Kazo, Y., Maehashi, M., Suzuki, S., Namba, E., Hoshiya, M., Hanai, R., Tozawa, Y., Morimoto, T., Ogasawara, N., Kageyama, Y., Ara, K., Ozaki, K., Yoshida, M., Kuroiwa, H., Kuroiwa, T., Ohashi, Y., and Kawamura, F. (2012). Expression of a small (p)ppGpp synthetase, YwaC, in the (p)ppGpp(0) mutant of *Bacillus subtilis* triggers YvyD-dependent dimerization of ribosome. *Microbiologyopen* 1**,** 115-134.

Takada, H., Fukushima-Tanaka, S., Morita, M., Kasahara, Y., Watanabe, S., Chibazakura, T., Hara, H., Matsumoto, K., and Yoshikawa, H. (2014). An essential enzyme for phospholipid synthesis associates with the *Bacillus subtilis* divisome. *Mol Microbiol* 91**,** 242-255.

Turnbull, K.J., Dzhygyr, I., Lindemose, S., Hauryliuk, V., and Roghanian, M. (2019). Intramolecular Interactions Dominate the Autoregulation of *Escherichia coli* Stringent Factor RelA. *Front Microbiol* 10**,** 1966.

Varik, V., Oliveira, S.R., Hauryliuk, V., and Tenson, T. (2016). Composition of the outgrowth medium modulates wake-up kinetics and ampicillin sensitivity of stringent and relaxed *Escherichia coli*. *Sci Rep* 6**,** 22308.
